# Freestanding Three-Dimensional CuO/NiO Core–Shell Nanowire Arrays as High-Performance Lithium-Ion Battery Anode

**DOI:** 10.1038/s41598-018-36378-0

**Published:** 2018-12-21

**Authors:** Yin-Wei Cheng, Chun-Hung Chen, Shu-Wei Yang, Yi-Chang Li, Bo-Liang Peng, Chia-Chin Chang, Ruey-Chi Wang, Chuan-Pu Liu

**Affiliations:** 1 Department of Materials Science and Engineering, National Cheng Kung University, Tainan, 70001 Taiwan; 2 Department of Greenergy, National University of Tainan, Tainan, 70005 Taiwan; 3 Department of Chemical and Materials Engineering, National University of Kaohsiung, Kaohsiung, 81148 Taiwan

## Abstract

We demonstrate significant improvement of CuO nanowire arrays as anode materials for lithium ion batteries by coating with thin NiO nanosheets conformally. The NiO nanosheets were designed two kinds of morphologies, which are porous and non-porous. By the NiO nanosheets coating, the major active CuO nanowires were protected from direct contact with the electrolyte to improve the surface chemical stability. Simultaneously, through the observation and comparison of TEM results of crystalline non-porous NiO nanosheets, before and after lithiation process, we clearly prove the effect of expected protection of CuO, and clarify the differences of phase transition, crystallinity change, ionic conduction and the mechanisms of the capacity decay further. Subsequently, the electrochemical performances exhibit lithiation and delithiation differences of the porous and non-porous NiO nanosheets, and confirm that the presence of the non-porous NiO coating can still effectively assist the diffusion of Li+ ions into the CuO nanowires, maintaining the advantage of high surface area, and improves the cycle performance of CuO nanowires, leading to enhanced battery capacity. Optimally, the best structure is validated to be non-porous NiO nanosheets, in contrary to the anticipated porous NiO nanosheets. In addition, considering the low cost and facile fabrication process can be realized further for practical applications.

## Introduction

Lithium-ion battery (LIB) is one of the dominant power sources for electric vehicles, medical devices, and personal electronics due to the long cycle life, high-energy density, and light weight. However, to meet the future high-energy demands needed by high-performance electric vehicles, electronics and medical devices, extensive research efforts have been directed toward the search of new anode materials of higher capacity as well as low-cost and long-term stability.

Thus far, graphite is the most common anode material, the theoretical capacity of which is limited to 372 mAh g^−1^. As such, various new anode materials have been studied for enhancing the performance of LIBs, such as CuO, NiO, Fe_2_O_3_, TiO_2_, SnO_2_, and Si^1–35^. Unfortunately, these anode materials have shortcomings as well, including low conductivity, excessive volume expansion during cycling, and low capacity, among others, thereby demanding more research. Among the above candidates, CuO stands out as probably the best compromise between high theoretical capacity, long cycle life and low cost for replacing graphite as the next generation anode material. Therefore, CuO has received extensive attention due to its relatively high theoretical capacity of 674 mAh g^−1^ (80% more than graphite), low raw materials cost and straightforward synthesis. Nevertheless, serious drawbacks for CuO remain, including problems in volume expansion and high irreversible insertion of lithium, which causes rapid degradation in capacity and cycle life. In response, many research groups have aimed at solving these problems by developing various heterogeneous nanostructures, nanowires, nanoparticles, and dendritic structures^2–14^.

Yet, CuO nanowires have been predominantly synthesized by a hydrothermal method at low temperatures, which offers the advantages of low cost but at the expense of crystallinity and adhesion^5,8,16^. The low crystallinity and poor adhesion leads to poor conductivity and LIB lifetime. Accordingly, attempts via annealing processes, additional conductive materials, and adhesive agents have been employed to respectively increase the crystallinity, conductivity, and adhesion to enhance LIB performance^1,3,9,15^. However, due to the formation of the unwanted Cu_2_O phase and the expensive process involved, capacity decay and high cost still remain. Consequently, CuO cannot be realized as a practical anode material until these problems are resolved.

In this paper, we first develop a facile process for synthesizing patterned CuO nanowires, with the aim of reducing the adverse effect of volume expansion; this is followed by coating the CuO nanowires with NiO nanosheets. NiO has several exciting advantages such as low cost, easy manufacturing, environmental friendliness, and high capacity. Several NiO nano-structures such as nanosheets, nanoparticles, nanomembranes, and nanofibers have been demonstrated with high reversible capacities even achieving 800–1000 mAh g^−1^ with favorable cycle life^36–42^. Therefore, the CuO nanowires with NiO nanosheets form a hierarchical structure to decrease the irreversible reactions while maintaining high capacity.

Subsequently, the dependence of porosity of NiO nanosheets on LIB performance is discussed, through which the optimal anode structure is demonstrated to be CnO nanowires coated with thin non-porous NiO nanosheets. The proposed method allows not only a stable solid-electrolyte interface layer to form, thereby benefitting to long cycle life, but also easy to implement, which is promising for mass production.

## Results and Discussion

**Figure 1:**
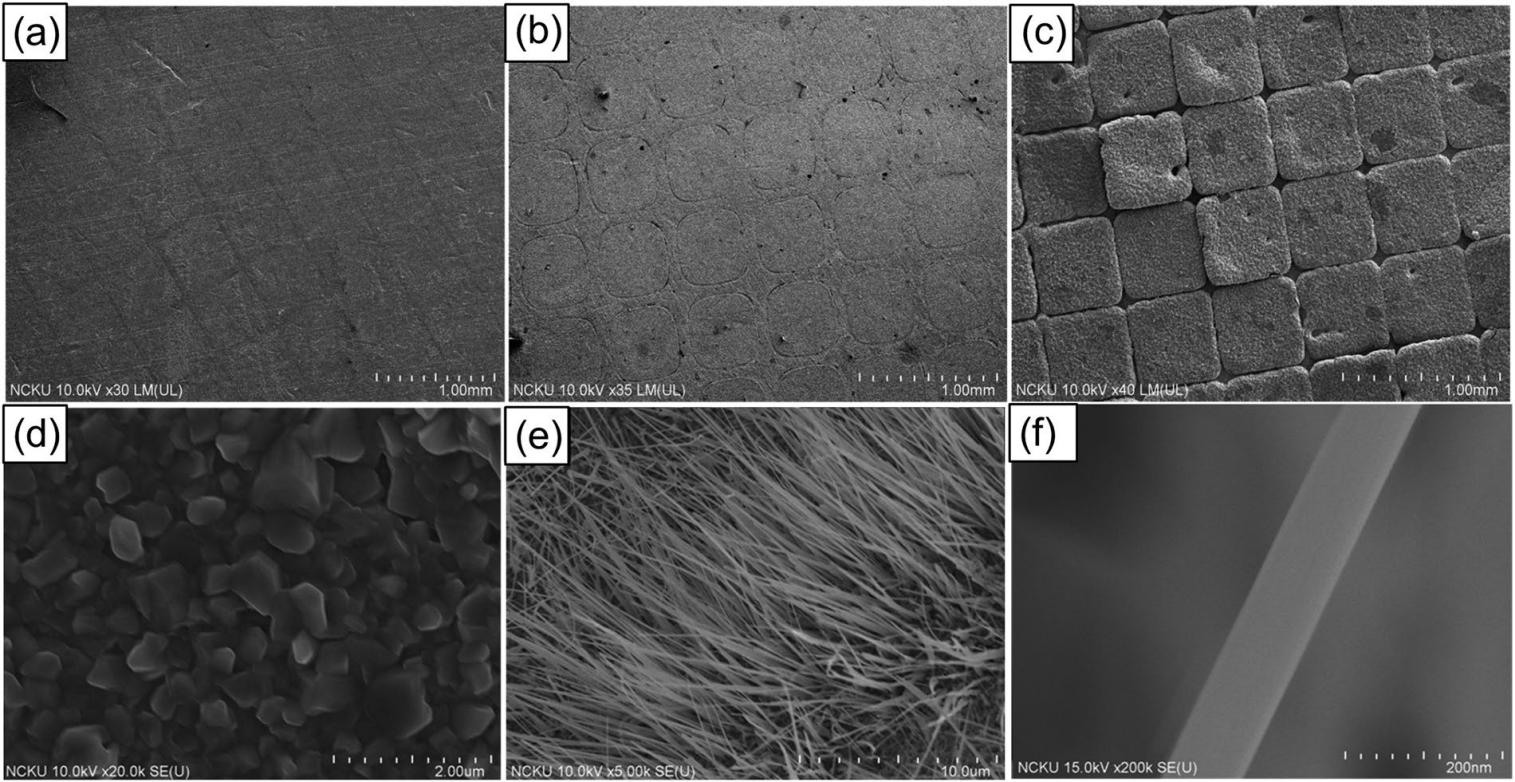
SEM images of (**a)** nickel pattern, (**b**) copper pattern, (**c**) CuO nanowire arrays pattern, (**d**) CuO nanoparticles in gap region, (**e**) CuO nanowires, and (**f**) close-up image of a single CuO nanowire in a grid region.

Upon thermal oxidation, the Cu film sequentially turned into Cu_2_O and CuO, and formed a CuO/Cu_2_O/Cu stacked film structure. Subsequently, single crystalline CuO nanowires emerged from the top of the stacked film via a stress-assisted growth mechanism. Nevertheless, it has been reported that the CuO layer easily peels off from the underlying Cu_2_O layer because of the presence of significant stress induced by the large mismatch in lattice constants and thermal expansion coefficients^43–47^. In the present work, we demonstrate the successful growth of large area CuO nanowire arrays with structural integrity by patterning. Figure 1a,b, present the SEM images of the Ni and Cu patterns, respectively. As can be seen, the pattern size remains almost the same after Cu electroplating, except that the pattern shape became rounded. Upon thermal oxidation, the patterns assist in effectively relieving the film stress by reserving a gap space for volume swelling, as shown in Fig. 1c, which prevents the CuO film with CuO nanowire arrays from peeling off. Moreover, the pattern is designed to achieve the maximum possible volume density of CuO nanowires without leaving any gaps during the CuO nanowires growth, which is evidenced by the grids nearly touching each other on the sides after volume swelling in Fig. 1c.

The distribution and morphology of the resulting CuO nanowires are examined in Fig. 1d–f of the SEM images. Figure 1e.f show that dense CuO nanowires are only grown on the top of the CuO/Cu_2_O/Cu stacked film. All the nanowires are rather dense, long and vertically aligned, as opposed to only CuO particles grown in the gap region shown in Fig. 1d. Figure 1f shows a typical CuO nanowire with a diameter between 60–80 nm. The grazing angle XRD patterns in the supporting information (Fig. S1) show that three phases coexist, including monoclinic CuO nanowires, a cubic-structure Cu_2_O film, and a face-centered-cubic Cu film, which together confirm the crystal phases of the stacked film. Additionally, CuO nanowires exhibit single and twin crystal structures, as shown in TEM images and diffraction patterns of Fig. S2 in the supporting information.

**Figure 2:**
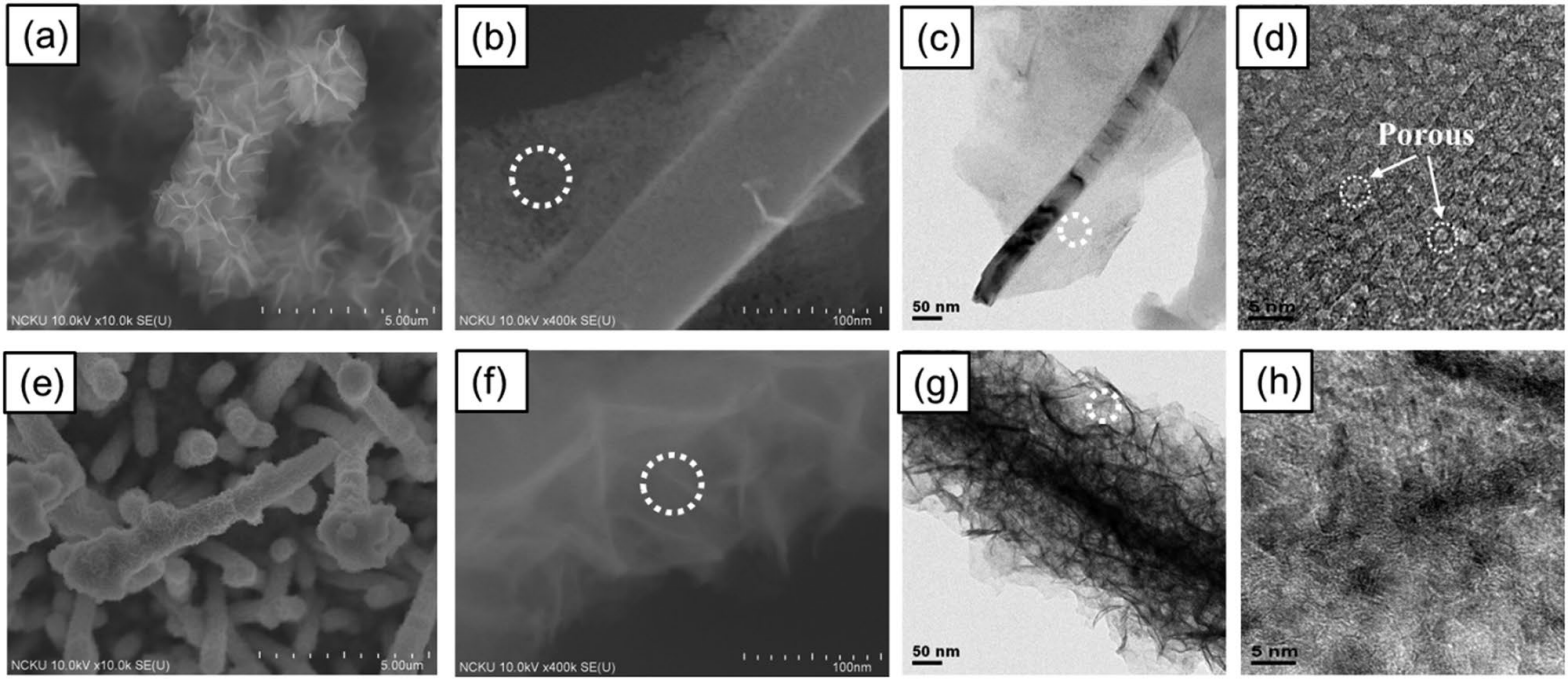
SEM images of the as-grown NiO nanosheets on the surface of the CuO nanowires by a hydrothermal method using precursors of (**a**–**d**) Ni(NO_3_)_2_, (**e**–**h**) NiSO_4_, forming different morphologies, where (**d**,**h**) are the HRTEM images of the white dotted circle in (**b**,**f**), exhibiting porous and non-porous structures, respectively.

**Figure 3:**
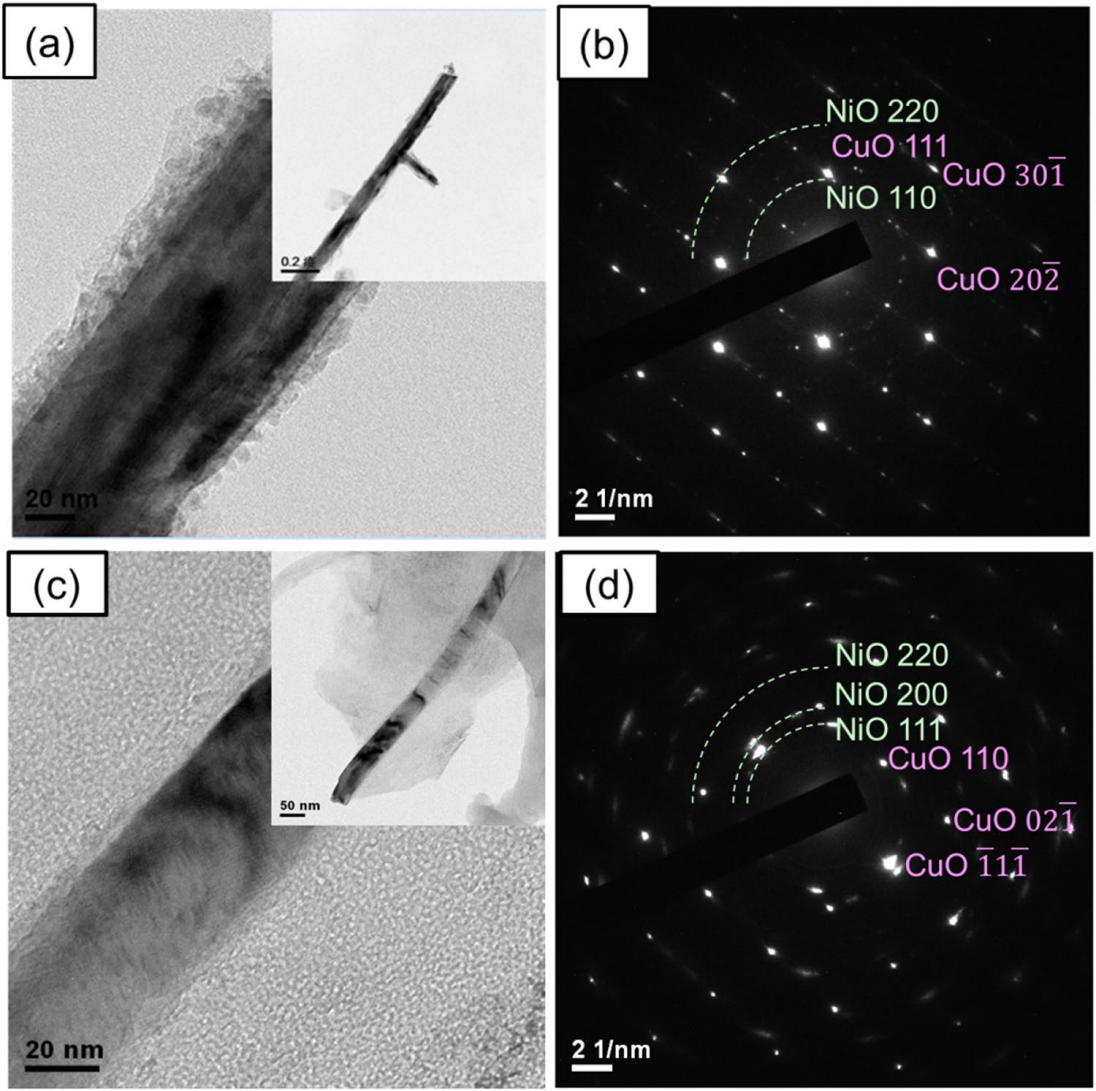
TEM analysis of the CuO/NiO(NiSO_4_) and CuO/NiO(Ni(NO_3_)_2_) nanowires before lithiation. (**a**) TEM images and (**b**) diffraction pattern of the CuO/NiO(NiSO_4_) hierarchical nanowires; (**c**) TEM images and (**d**) diffraction pattern of the CuO/NiO(Ni(NO_3_)_2_) hierarchical nanowires.

NiO nanosheets were grown on the surface of the CuO nanowires via a hydrothermal method. On examining the morphologies of the as-grown NiO nanosheets by SEM and TEM in Fig. 2, it can be seen that two drastically different morphologies were formed. As shown in Fig. 2a,c, bigger pieces of NiO nanosheets completely cover the surfaces of bundles of CuO nanowires by using Ni(NO_3_)_2_ precursors, where individual NiO nanosheets are composed of densely populated tiny pores measuring a few nanometers, as revealed in the HRTEM images of Fig. 2b,d by slight defocus. By contrast, when using NiSO_4_ precursors, the NiO nanosheets form smaller pieces and conformally coat the surface of individual CuO nanowires, as shown in Fig. 2e,g, where individual NiO nanosheets are non-porous, as shown in Fig. 2f and h. Before lithiation, the TEM images and diffraction patterns of CuO/NiO (Ni(NO_3_)_2_) and CuO/NiO(NiSO_4_) nanowires as shown in Fig. 3a–d indicate no Cu_2_O present in these nanowires. The STEM EDX maps in Fig. S3 of the supporting information reveal clearly that CuO and NiO are distributed in the cores and surfaces, respectively, of the CuO/NiO(NiSO_4_) and CuO/NiO(Ni(NO_3_)_2_ nanowires.

During synthesis, smaller NO^3−^ molecules can more easily intercalate inside the nanosheets than larger SO_4_^2−^ molecules, which leads to the formation of tiny pores by the evaporation of the NO^3−^ molecules upon subsequent thermal annealing^48^. Growth of the NiO phase in both the CuO/NiO(NiSO_4_) and CuO/NiO(Ni(NO_3_)_2_ nanosheets is clearly observed in the XRD patterns in Fig. S4 of the supporting information. According to the results of the TEM diffraction patterns in Fig. 3, the Cu_2_O peaks in the XRD patterns of S4 should originate from the remaining Cu_2_O film underneath instead of the CuO/NiO nanowires. For comparison, NiO nanosheets were also grown on only a pure stainless steel substrate without CuO nanowires by using NiSO_4_ precursors. As shown in the SEM images of different magnifications in the supporting information (Fig. S5a–d), the substrate is covered by spheroidic particles, which are composed of self-assembled NiO nanosheets, in close resemblance to the morphology of the NiO nanosheets of the CuO/NiO nanowires in Fig. 2f. However, the poor contact leads to incomplete coverage on the stainless steel substrate shown in Fig. S5a.

**Figure 4:**
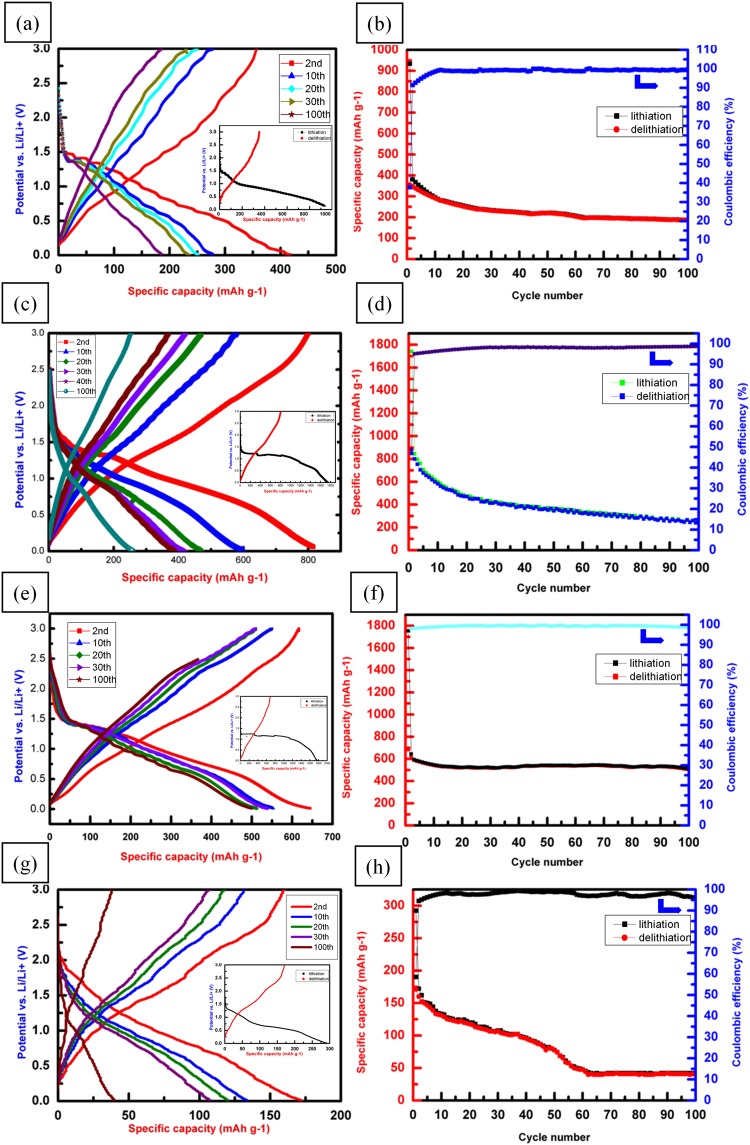
Electrochemical performances of LIBs using (**a**,**b**) pure CuO, (**c**,**d**) CuO/NiO(Ni(NO_3_)_2_), (**e**,**f)** CuO/NiO(NiSO_4_) nanowires, and (**g**,**h**) NiO(NiSO_4_) sheets as anode electrodes. Please note that (**a**,**c**,**e**,**g**) show potentials and capacities of the lithiation/delithiation cycles, while (**b**,**d**,**f**,**h**) show lithiation/delithiation capacities and coulombic efficiencies for 100 cycles. The insets of (**a**,**c**,**e**,**g**) show the electrochemical performances of the first lithiation/delithiation cycle.

The electrochemical performances of the LIB coin cells made of CuO, CuO/NiO(Ni(NO_3_)_2_), CuO/NiO(NiSO_4_), and NiO(NiSO_4_) nanowire arrays are compared in Fig. 4. The CuO nanowires coin cell, shown in Fig. 4a,b exhibits the typically fast-fading behaviors in cycle retention along with the fast-disappearing voltage plateau during the lithiation and delithiation cycles, although reasonably high capacity can be initially achieved. The capacity decay is caused by two mechanisms. First, the CuO nanowires suffer from volume expansion/contraction during periodic lithiation/delithiation leading to the formation of fractures, which may allow the electrolyte to permeate through the cracks inside the nanowires and form fresh solid-electrolyte-interphase (SEI) layers. The SEI may further induce more stress^49,50^, thereby facilitating further facture of the CuO nanowires. Second, owing to the incomplete oxidation reaction of Cu during the delithiation process, lower capacity Cu_2_O gradually forms in replace of high capacity CuO^14^. The CuO LIB performance can be significantly improved by coating NiO nanosheets in both cases of the CuO/NiO(Ni(NO_3_)_2_) and CuO/NiO(NiSO_4_), as shown in Fig. 4c–f. The overall reactions of CuO and NiO during lithiation/delithiation are given by Eqs (1) and (2)^8,51^:

1 $${\rm{CuO}}+2{{\rm{Li}}}^{+}+2{{\rm{e}}}^{-}\leftrightarrow {{\rm{Li}}}_{2}{\rm{O}}+{\rm{Cu}}$$

2 $${\rm{NiO}}+2{{\rm{Li}}}^{+}+2{{\rm{e}}}^{-}\leftrightarrow {\rm{Ni}}+{{\rm{Li}}}_{2}{\rm{O}}$$

Equation (1) combines multistep electrochemical reactions as given below:

3 $${\rm{CuO}}+{{\rm{xLi}}}^{+}+{{\rm{xe}}}^{-}\to {{\rm{Cu}}}_{1-{\rm{x}}}^{{\rm{II}}}{{\rm{Cu}}}_{{\rm{x}}}^{{\rm{I}}}{{\rm{O}}}_{1-{\rm{x}}/2}+{\rm{x}}/2{{\rm{Li}}}_{2}{\rm{O}}\,(0 < {\rm{x}} < 0.4)$$

4 $${{\rm{Cu}}}_{{\rm{1}}-{\rm{x}}}^{{\rm{II}}}{{\rm{Cu}}}_{{\rm{x}}}^{{\rm{I}}}{{\rm{O}}}_{1-{\rm{x}}/2}+(1-{\rm{x}}){{\rm{Li}}}^{+}+(1-{\rm{x}}){{\rm{e}}}^{-}\to 1/2{{\rm{Cu}}}_{2}{\rm{O}}+(1-{\rm{x}})/2{{\rm{Li}}}_{2}{\rm{O}}$$

5 $$1/2{{\rm{Cu}}}_{2}{\rm{O}}+{{\rm{Li}}}^{+}+{{\rm{e}}}^{-}\to {\rm{Cu}}+1/2{{\rm{Li}}}_{2}{\rm{O}}$$

The first step (Eq. 3) shows that Li reacts with CuO to forms the intermediate composite copper oxide phase. In the second step (Eq. 4), the reduction reaction leads to Cu_2_O formation. Ultimately, Cu_2_O is decomposed into Cu and Li_2_O (Eq. 5)^8^. In the initial lithiation/delithiation curves of the CuO/NiO anodes, we can observe two lithiation plateaus around 1.1–1.25 V and 0.5–0.75 V and two vague delithiation plateaus around 1.3 and 2.3 V in the CuO/NiO(NiSO_4_). The lithiation plateaus represent insertion of lithium into CuO and NiO, and the reduction of Cu^2+^ and Ni^2+^ into their metallic states with the formation of Li_2_O. The delithiation platforms around 1.3 and 2.3 V can be attributed to the oxidation reactions of metallic Cu and Ni, respectively^52,53^.

In order to further understand the lithiation/delithiation mechanism, Fig. S6 of the supporting information presents the CV curves of sample CuO, CuO/NiO(Ni(NO_3_)_2_), CuO/NiO(NiSO_4_), and NiO(NiSO_4_) for the first three cycles at a scan rate of 0.1 mV s^−1^ in the potential range of 0–3.0 V^54–56^.

In comparing the attainable maximum capacity after the first cycle, CuO/NiO(Ni(NO_3_)_2_) is higher at 821 mAh g^−1^, followed by 617 mAh g^−1^ for the CuO/NiO(NiSO_4_) nanowires, both are closer to their theoretical capacities and outperform the 400 mAh g^−1^ of pure CuO. This indicates that two types of NiO nanosheets not only contribute to part of the capacity, but also protect the core CuO nanowires from degradation by boosting the chemical stability of the surfaces. However, the porous NiO nanosheets provide the fastest ionic conduction paths through the numerous pores, thereby achieving the highest capacity as confirmed in Fig. S7 of the supporting information. In addition, the cycle retention was observed to sustain for 100 cycles at 0.1C rate, which can be ascribed to the effective relaxation of macrostress to maintain integrity over the entire structure. Nevertheless, the fast-fading phenomenon found in the CuO nanowires battery was drastically reduced by the CuO/NiO(NiSO_4_) nanowires from 617 mAh g^−1^ to 522 mAh g^−1^ at 84.6% retention for 100 cycles with the best coulombic efficiency at around 98%, as compared to 30.8% retention from 821 mAh g^−1^ to 253 mAh g^−1^ for the CuO/NiO(Ni(NO_3_)_2_) battery. Therefore, the conformal coating of continuous, yet thin NiO nanosheets on the surface of the CuO nanowires promises the best route to protect them from chemical and mechanical degradation while still boosting effective capacity. The hierarchical CuO/NiO nanowires play an important role in achieving such excellent battery performance with the CuO nanowires, which is evidenced by the poor battery performance of NiO(NiSO_4_) nanosheets alone, as shown in Fig. 4g,h, including low capacity and poor retention likely due to the poor contact with the stainless steel substrate (see Fig. S5a).

**Figure 5:**
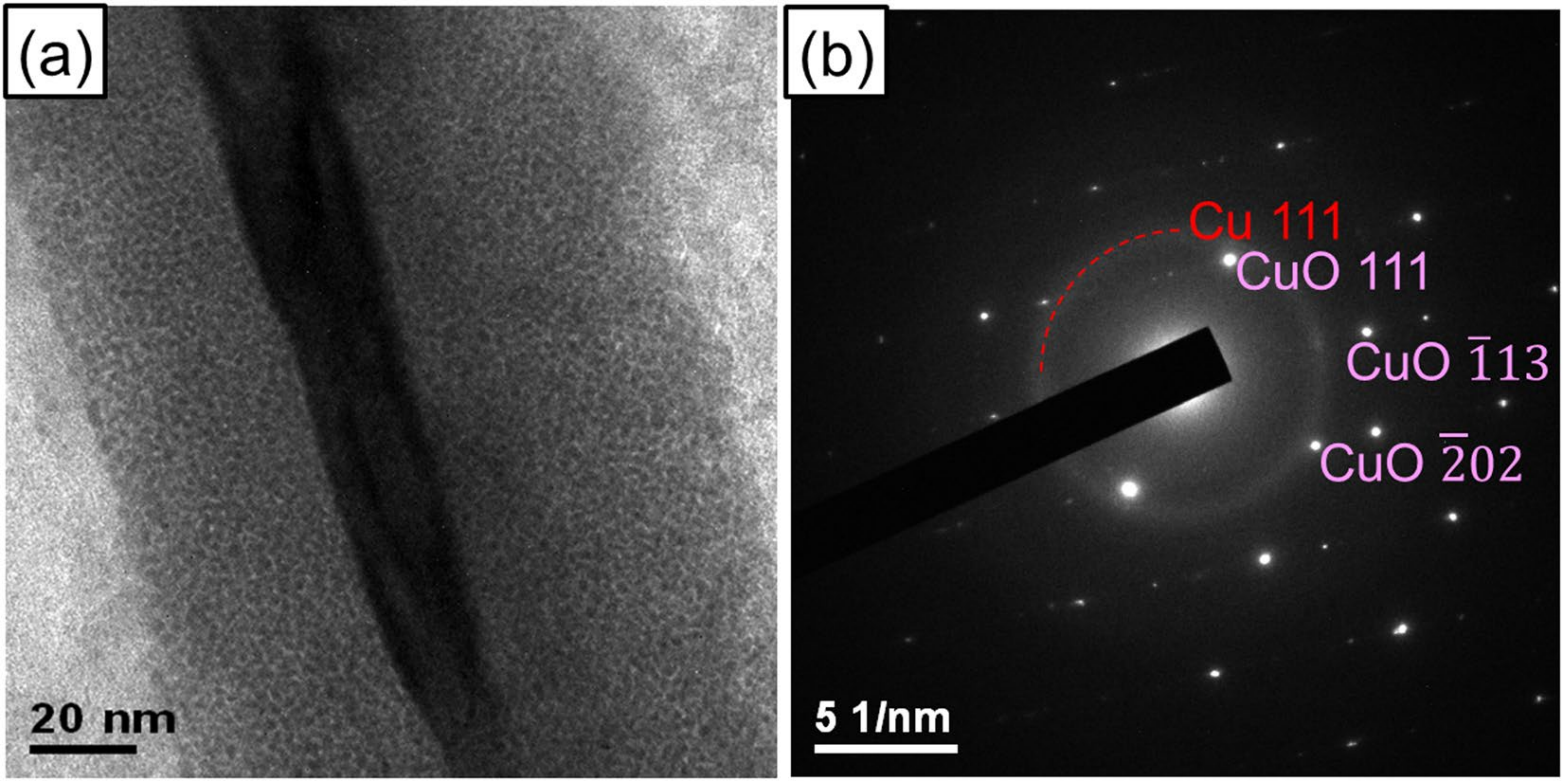
TEM analysis of the uncoated CuO nanowires upon reactions with Li ions during the lithiation process of the first cycle: (**a**) TEM image, and (**b**) diffraction pattern.

The mechanism for the improvement of capacity and retention of the CuO nanowires as anode materials by coating with NiO nanosheets was investigated in depth by TEM. As shown in Fig. 5, the CuO nanowire has expanded in size by approximately two times upon the lithiation process at the initial cycles. At this stage, the highly crystalline CuO nanowire has transformed into a core/shell structure. Whereas the outer shell region appears to be non-crystalline, representing the range of chemical reactions during lithiation, the highly-crystalline core is apparently an unreacted region of only about 20 nm in size. The diffraction pattern in Fig. 5b further reveals that tiny Cu clusters are uniformly imbedded inside an amorphous matrix, which is possibly a mixture of Li_2_O and other chemical ingredients from the electrolyte to form an SEI phase. The complex phases in the amorphous shell could impede the lithium ions from deeper diffusion, limiting the attainable capacity and leading to the higher irreversible capacity in the first cycle as well as fast capacity fading in the subsequent cycles.

**Figure 6:**
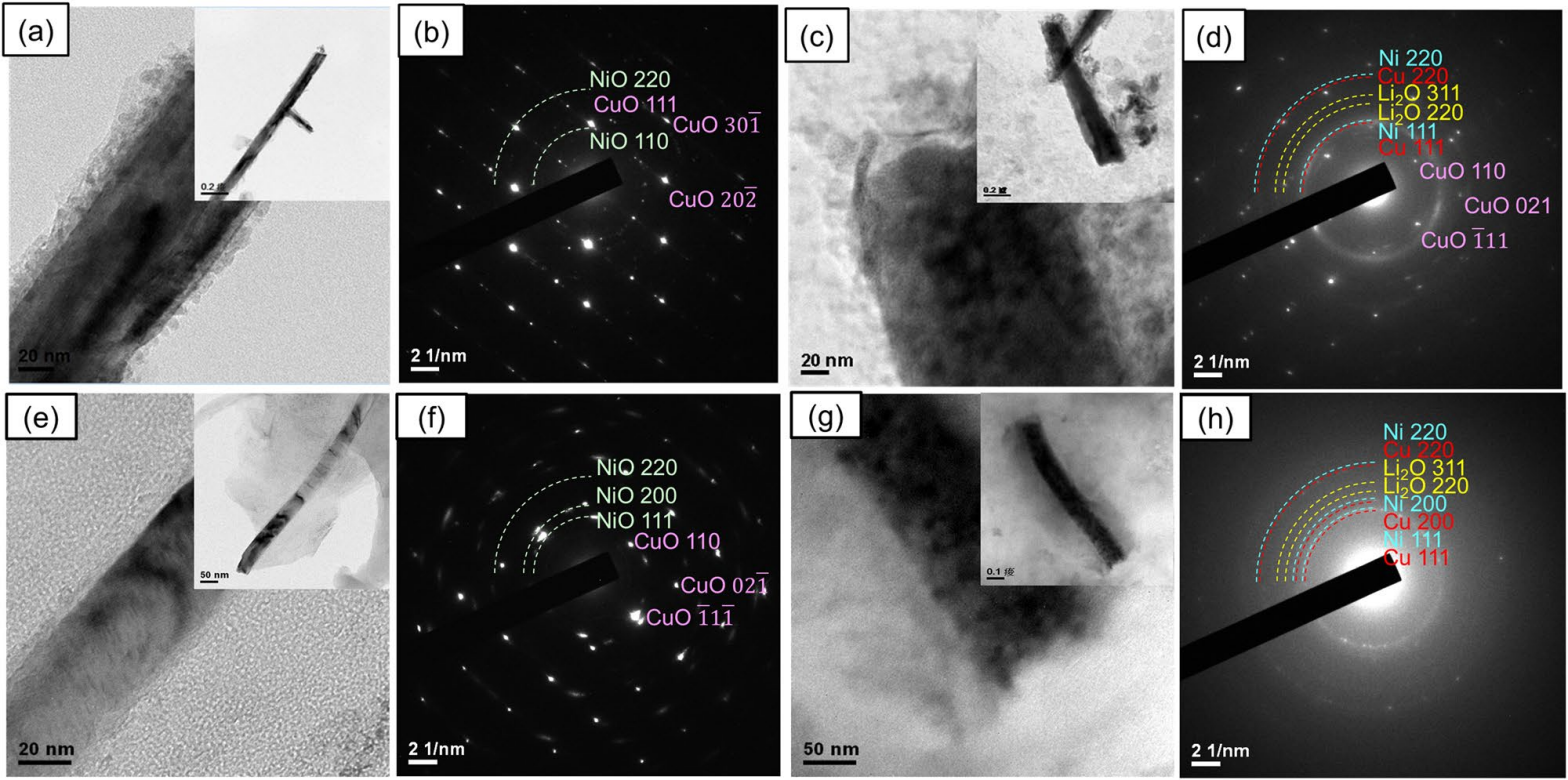
TEM analysis of the CuO/NiO(NiSO_4_) and CuO/NiO(Ni(NO_3_)_2_) nanowires before and after lithiation. (**a**–**d**) and (**e**–**h**) are for the CuO/NiO(NiSO_4_) and CuO/NiO(Ni(NO_3_)_2_) hierarchical nanowires, respectively, where (**a**,**e**) TEM images and (**b**,**f**) diffraction patterns before lithiation; (**c**,**g**) TEM images and (**d**,**h**) diffraction patterns upon the first cycle lithiation.

Figure 6a–h examine the microstructure evolution of the CuO/NiO(NiSO_4_) and CuO/NiO(Ni(NO_3_)_2_) nanowires respectively before and after lithiation. Consistent with the SEM images, Fig. 6a,e show that the CuO nanowires are coated with a layer of NiO nanosheets before electrochemical reactions. Figure 6b,f also confirm that the NiO nanosheets are polycrystalline and the core CuO nanowire exhibits a single crystalline structure. During the lithiation process for the first cycle, Fig. 6c,g show that the CuO nanowires expand by 2 times to 220 nm and 3 times to 110 nm in the CuO/NiO(NiSO_4_) and CuO/NiO(Ni(NO_3_)_2_), respectively. The entire core CuO region has transformed into Cu and Li_2_O, as confirmed by the diffraction patterns in Fig. 6d,h resulting from the lithiation of CuO, and still predominantly remains crystalline with a clear interface with the shell. Nevertheless, the presence of CuO of the CuO/NiO(NiSO_4_) nanowire after the first lithiation cycle as in Fig. 6d indicates not achieving full lithiation. The outer shell region has also expanded and has become amorphous, composed of a mixture of Ni and SEI phases. In contrast with CuO/NiO(NiSO_4_), Fig. 6h indicates that CuO/NiO(Ni(NO_3_)_2_) nanowire achieves full lithiation upon the first lithiation cycle due to the absence of CuO. Therefore, the difference of crystallinity is one of the causes in the different performance between CuO/NiO(Ni(NO_3_)_2_) and CuO/NiO(NiSO_4_).

The TEM results suggest that the NiO nanosheets can isolate the active CuO nanowires from reactions with the electrolyte, unlike the case of the uncoated CuO nanowires, to form a rather stable protective layer. Simultaneously, the protective layer is thin enough for the conduction of lithium ions, allowing lithiation and delithiation of active CuO nanowires without any side chemical reactions with other chemical species diffused from the electrolyte. Moreover, the NiO nanosheets did not crack in the CuO/NiO(NiSO_4_) nanowire and may provide a buffer for reducing the volume expansion of the CuO nanowire in the core. Accordingly, to the best of our knowledge, this novel structure of CuO/NiO(NiSO_4_) scheme facilitates the active CuO anode materials to approach the theoretical capacity with minimal capacity decay even up to 100 cycles, which demonstrates the best performance of active CuO as anode materials that can be achieved for LIBs. Table 1 summarizes the material morphologies, crystal suructures, and electrochemical performances of the CuO, CuO/NiO(Ni(NO_3_)_2_), CuO/NiO(NiSO_4_), and NiO(NiSO_4_) anode electrodes for comparison.

**Table 1:** Comparison of material morphology, crystal structure and electrochemical performance between CuO, CuO/NiO(Ni(NO_3_)_2_), CuO/NiO(NiSO_4_), and NiO(NiSO_4_) anode electrode.

Material	CuO	CuO/NiO(Ni(NO_3_)_2_)	CuO/NiO(NiSO_4_)	NiO(NiSO_4_)
Morphology	Nanowires	Porous hierarchical Nanowires	Hierarchical nanowires	Nanosheets
Crystal Structure	Single crystal	Hierarchical	Hierarchical	Polycrystal
Capacity 2nd (mah g^−1^)	400	821	617	160
Capacity 100th (mah g^−1^)	192	253	522	40
Capacity Retention	48%	30.8%	84.6%	25%

## Methods

### Fabrication process of high crystalline CuO nanowire arrays and NiO nanosheets

**Figure 7:**
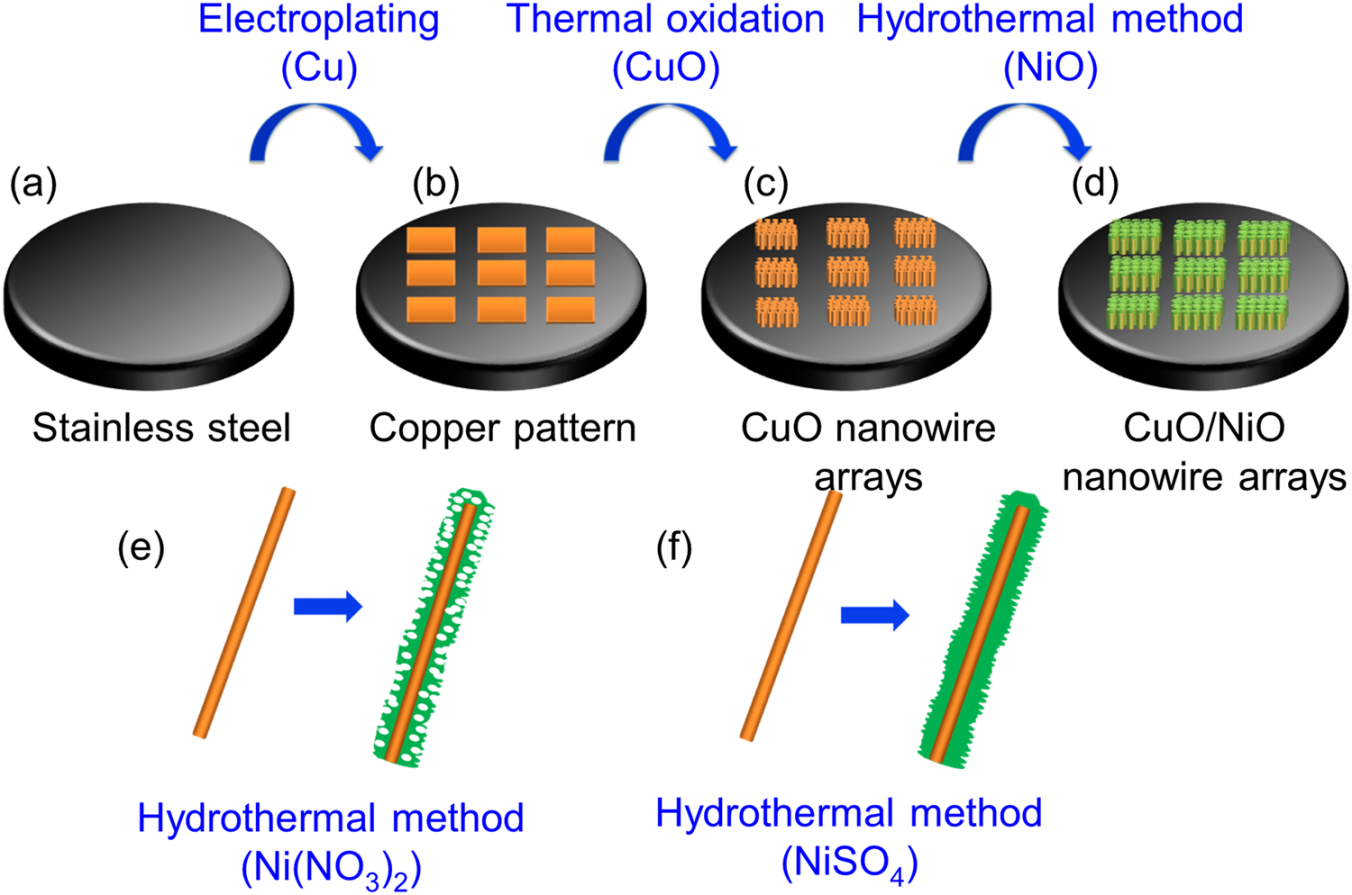
Schematic diagrams of the procedure for preparing CuO/NiO nanowire arrays: (**a**) preparing the stainless steel substrate, (**b**) making the square Cu patterns, (**c**) growth of the CuO nanowire arrays by thermal oxidation, and (**d**) growth of the NiO sheets as NiO/CuO core/shell structure by a hydrothermal method, where individual nanowires form (**e**) porous and (**f**) non-porous shells when Ni(NO_3_)_2_ or NiSO_4_ is employed as the precursor, respectively.

Figure 7 illustrates the entire fabrication process of the CuO/NiO nanowires for the experiment. Patterned Ni square arrays of 508 µm in each pitch, consisting of a hole width of 425 µm, bar width of 83 µm, and a thickness of 30 nm were first deposited on a stainless steel sheet by precision etching and coating system (PECS) with a shadow mask of stainless steel grids. Subsequently, a 2 μm thick Cu film was selectively grown on top of the Ni grids by Cu electroplating (Fig. 7b). Next, the CuO nanowire arrays were formed by oxidizing the Cu film via thermal annealing at 400 °C for 4 hours (Fig. 7c). Finally, NiO nanosheets were coated on the surface of the CuO nanowires by a hydrothermal method (Fig. 7d), in which the stainless steel sheet with CuO nanowire arrays was immersed in a 0.03 M mixed solution and kept in a water bath at 85 °C for 1 hour followed by annealing at 350 °C for 2 hours. Figure 7e,f, show that pores may or may not form in the NiO nanosheets when Ni(NO_3_)_2_ and NiSO_4_ was mixed with hexamethylenetetramine (HMTA) in the solution, respectively.

### Structural characterization and electrochemical measurements

The microstructure, morphology and crystallinity of the as-prepared CuO and CuO/NiO nanowires were analyzed by transmission electron microscopy (TEM, JEM-2100F CS STEM, JEOL, Japan), scanning electron microscopy (SEM, SU8000, JEOL, Tokyo, Japan), and X-ray diffraction (XRD), respectively. The CuO/NiO nanowires were assembled into coin cells as the anode for testing LIB performance. The assembling sequence from bottom to top is as follows: bottom stainless steel case, Li metal cathode, separator, CuO/NiO nanowires, spring, and top stainless steel case; the electrolyte is ethylene carbonate (EC)/diethyl carbonate (DEC) with 1 M LiPF6. The cycle tests of the anode materials were performed between 0.1–3 V under a constant current of 0.1 C (70 mA g^−1^) at room temperature. For comparison, evolution of the morphology and microstructure of the CuO/NiO nanowires upon cycling were studied by TEM, where a constant current of 1 µA was employed for the formation process in the 1st cycle by Keithley 2400.

## Electronic supplementary material

Freestanding Three-Dimensional CuO/NiO Core–Shell Nanowire Arrays as High-Performance Lithium-Ion Battery Anode
