# Supplementary material for: Freestanding Three-Dimensional CuO/NiO Core–Shell Nanowire Arrays as High-Performance Lithium-Ion Battery Anode
Source: Sci Rep. 2018 Dec 21;8:18034. doi: 10.1038/s41598-018-36378-0 (PMC6303307; doi:10.1038/s41598-018-36378-0)
Supplement: Supplementary file 1 — Freestanding Three-Dimensional CuO/NiO Core–Shell Nanowire Arrays as High-Performance Lithium-Ion Battery Anode [file 41598_2018_36378_MOESM1_ESM.docx]

**Electronic Supplementary Material**

**Freestanding Three-Dimensional CuO/NiO Core–Shell Nanowire Arrays as High-Performance Lithium-Ion Battery Anode**

**Yin-Wei Cheng**^1^**, Chun-Hung Chen**^1^**, Shu-Wei Yang**^1^**, Yi-Chang Li**^1^**, Bo-Liang Peng**^1^**, Chia-Chin Chang**^2^**, Ruey-Chi Wang**^3^**, and Chuan-Pu Liu**^1,*^

^1^Department of Materials Science and Engineering, National Cheng Kung University, Tainan, 70001, Taiwan

^2^Department of Greenergy, National University of Tainan, Tainan, 70005, Taiwan

^3^Department of Chemical and Materials Engineering, National University of Kaohsiung, Kaohsiung, 81148, Taiwan

*cpliu@mail.ncku.edu.tw

**S1. Grazing angle X-ray diffraction (XRD) pattern of CuO nanowire arrays**

According to the XRD pattern, three phases coexist, namely monoclinic CuO nanowires, Cu_2_O film of the cubic structure, and Cu film of the face-centered cubic structure. We can observe that all the higher intensity peaks correspond to CuO, implying that CuO nanowires occupy a large volume fraction. The presence of tiny Cu peaks indicates that residual Cu metal still remains, and has not oxidized completely. Also, small peaks of Cu_2_O confirm that the growth of CuO nanowires starts from the formation of a thin layer of Cu_2_O.


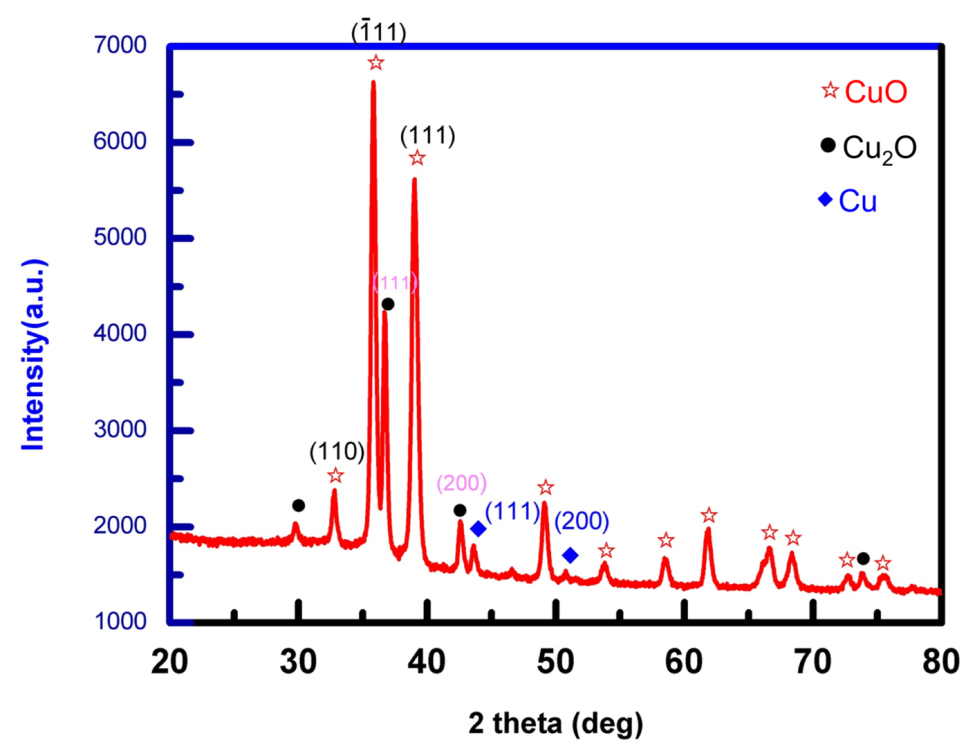


**Figure S1** XRD pattern of CuO nanowire arrays.

**S2. TEM images and diffraction patterns of CuO nanowire arrays as single and twin crystal structures**

According to the diffraction patterns of figures S2(a) and S2(b), we can see the direction of (-1 1 0) is perpendicular to the edge of nanowire. Fig. S2(b) shows CuO nanowire is on [0 0 1] zone axis and we can judge that the growth direction of CuO nanowire must be both perpendicular (-1 1 0) and [001]. Therefore, the growth direction is parallel to [ -7 -7 -1] (Growth direction : [0 0 1] × (-1 1 0)//[ -7 -7 -1] ), consisting of the two-dimensional defects of stacking faults represented by dark and bright fringes. On the other hand, Figs. S2(c) and (d) clearly show the twinned structure in the CuO nanowire with the twinning plane of (-1 -1 -3).


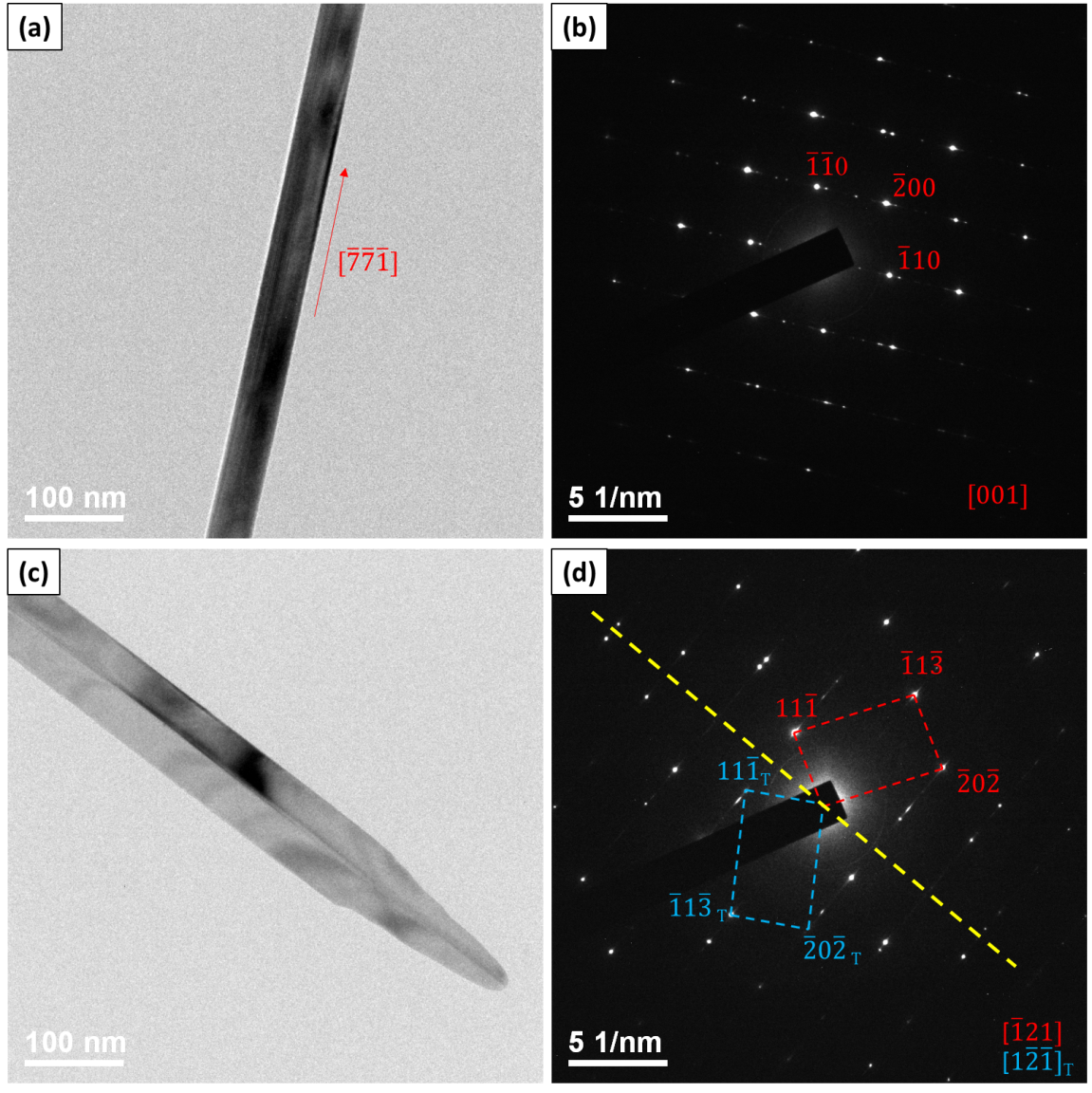


**Figure S2** TEM analysis of the single and twin structures in the CuO nanowire: a) and b) are the bright-field low-magnification image and diffraction pattern of a single nanowire, respectively, while c) and d) are the respective bright-field low-magnification image and diffraction pattern of a twinned nanowire.

**S3. EDX mappings of CuO/NiO(NiSO_4_) and CuO/NiO(Ni(NO_3_)_2_ nanowires**

The EDX mappings of STEM in Fig. S3 reveal clearly that the CuO and NiO are distributed respectively in the cores and surfaces of the CuO/NiO(NiSO_4_) and CuO/NiO(Ni(NO_3_)_2_ nanowires.


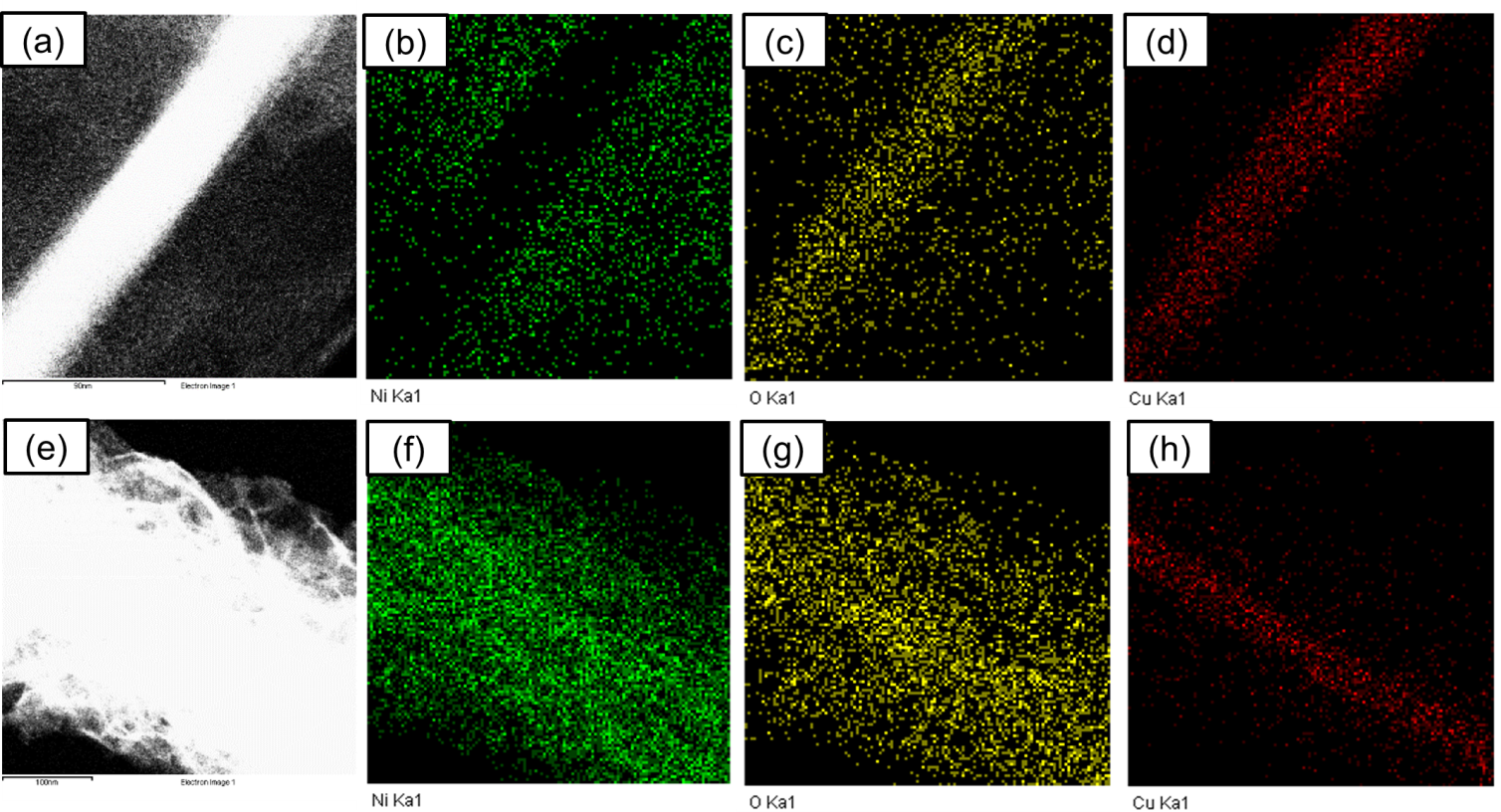


**Figure S3** EDX maps of CuO/NiO(NiSO_4_) and CuO/NiO(Ni(NO_3_)_2_) nanowires. a) – d) STEM and EDX images of CuO/NiO(Ni(NO_3_)_2_) nanowire; e) – f) STEM and images of CuO/NiO(NiSO_4_) nanowire; a) and e) are STEM images, b) and f) are Ni maps, c) and g) are O maps, and d) and h) are Cu maps.

**S4. XRD patterns of CuO/NiO(NiSO_4_) and CuO/NiO(Ni(NO_3_)_2_ nanosheets**

The XRD patterns in Figure S4 reveal that the NiO nanosheets in the NiO(NiSO_4_) spheroidals are polycrystalline with the Fe peaks originating from the stainless steel foil. Nevertheless, both the CuO/NiO(NiSO_4_) and CuO/NiO(Ni(NO_3_)_2_) samples exhibit the strongest peaks corresponding to CuO with minor Cu_2_O and only minimal signals from NiO (200). Due to the low-temperature process, the crystallinity of NiO is much poorer than CuO.

**
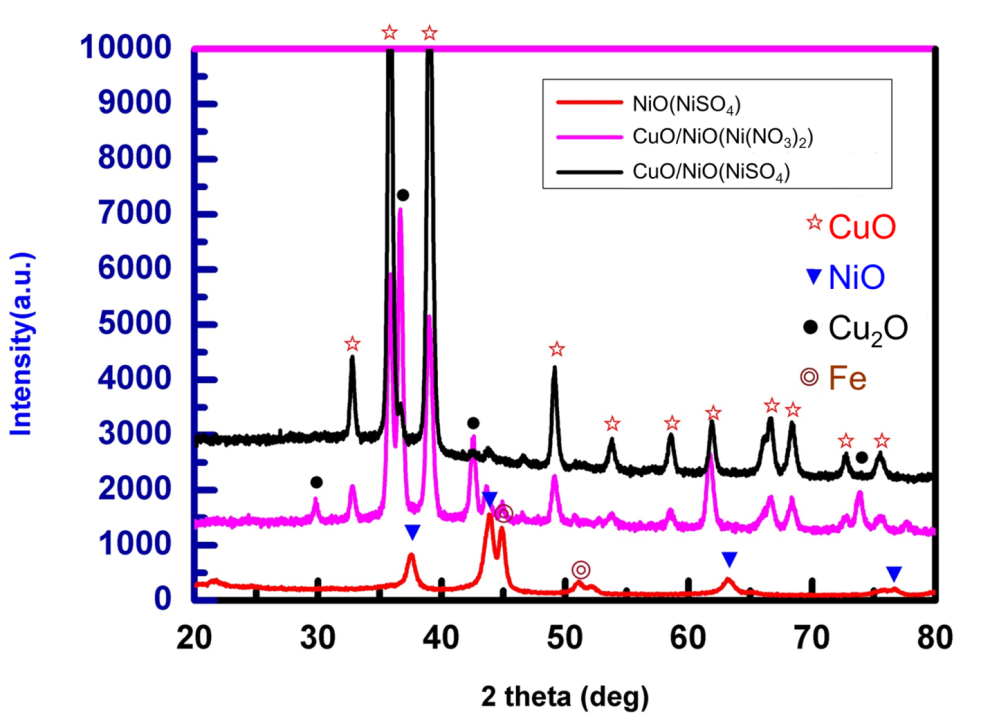
**

**Figure S4** XRD patterns of CuO/NiO(NiSO_4_) nanowires, CuO/NiO(Ni(NO_3_)_2_) nanowires, and the spheroidic NiO(NiSO_4_) nanosheets on the stainless steel foil.

**S5. SEM images of the NiO nanosheets grown on pure stainless steel foil**

The NiO nanosheets were also grown on only a pure stainless steel substrate without CuO nanowires by using NiSO4 precursors. As shown in the SEM images of different magnifications in Figs S4a-d, the substrate is covered by spheroidic particles, which are composed of self-assembled NiO nanosheets. In addition, the poor contact leads to incomplete coverage on the stainless steel substrate.


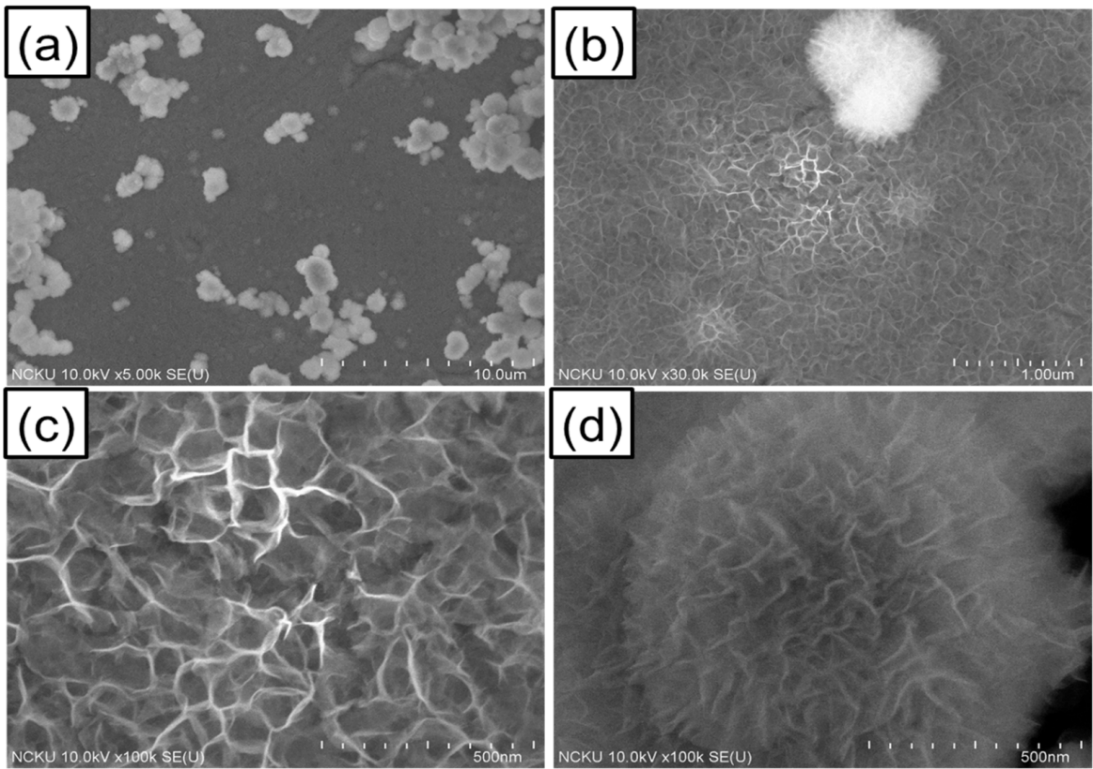


**Figure S5** SEM images of the NiO nanosheets grown on pure stainless steel foil. (a) – (d) SEM images of different magnifications, showing that the morphology is composed of spheroidic particles.

**S6. The CV curves of CuO, CuO/NiO(Ni(NO_3_)_2_), CuO/NiO(NiSO_4_), and NiO(NiSO_4_)**

The fig. 6Sa shows the CV curves of the CuO sample for three cycles. In the first cathodic scan, there are two peaks including a small peak at 1.36 V and a broad peak at 0.6-1.0 V, corresponding to the first reduction to Cu_2_O and further reduction to Cu accompanied by the formation of Li_2_O. In the first anodic scan, there are three broad peaks located at 0.6-1.0 V, 1.0-1.7 V and 2.3-2.65 V, respectively, where the main anodic broad peak at 1.0-1.7 V and 2.3-2.65 V are ascribed to oxidation reaction from Cu to Cu_2_O and finally oxidation into CuO, respectively, and the 0.6-1.0 V peak could be associated with decomposition of the SEI layer.^54, 55, 56^ In the subsequent cycles, the cathodic peaks undergo slight shift and become narrower. Besides, the decreases in the intensity and integral area of individual peaks with the number of cycles indicate reversible capacity losses.

Fig. S6b and S6c show the CV curves of sample CuO/NiO(Ni(NO_3_)_2_) and CuO/NiO (NiSO_4_), respectively, and they behave differently from sample Cu. In the first cathodic scan, they both have two broad peaks, located at (1.34-1.57 V and 0.76-1.25 V) and (0.80-1.42 V and 0.51-0.74 V) correspondingly, resulting from the reduction reactions of Cu^2+^ and Ni^2+^ to metallic Cu and Ni with the formation of Li_2_O. In addition, an additional peak observed at 0-0.5 V could be ascribed to the formation of solid electrode interface (SEI) formation. In the first anodic scan, two broad peaks of CuO/NiO(Ni(NO_3_)_2_) and CuO/NiO (NiSO_4_) are located at (1.15-1.88 V and 2.06-2.56 V) and at (0.82-1.16 V and 2.0-2.7 V), respectively, resulting from oxidation into CuO and NiO. Besides, one slightly stronger anodic peak in each spectrum between 0.5 and 1.0 V could be attributed to the decomposition of the SEI layer.

For comparison, the CV curve of the NiO(NiSO_4_) sample is shown in Fig. S6d. In the first cathodic scan, two peaks are located at 0.4-1.0 V and 1.03-1.32 V, resulting from the reduction into Ni with the formation of Li_2_O. In the anodic scan, three peaks are observed where the first two peaks located at 0.81-1.14 V and 1.15-1.72 V can be attributed to the decomposition of the SEI layer and the last peak at 1.90-2.81 V results from the oxidation into NiO.


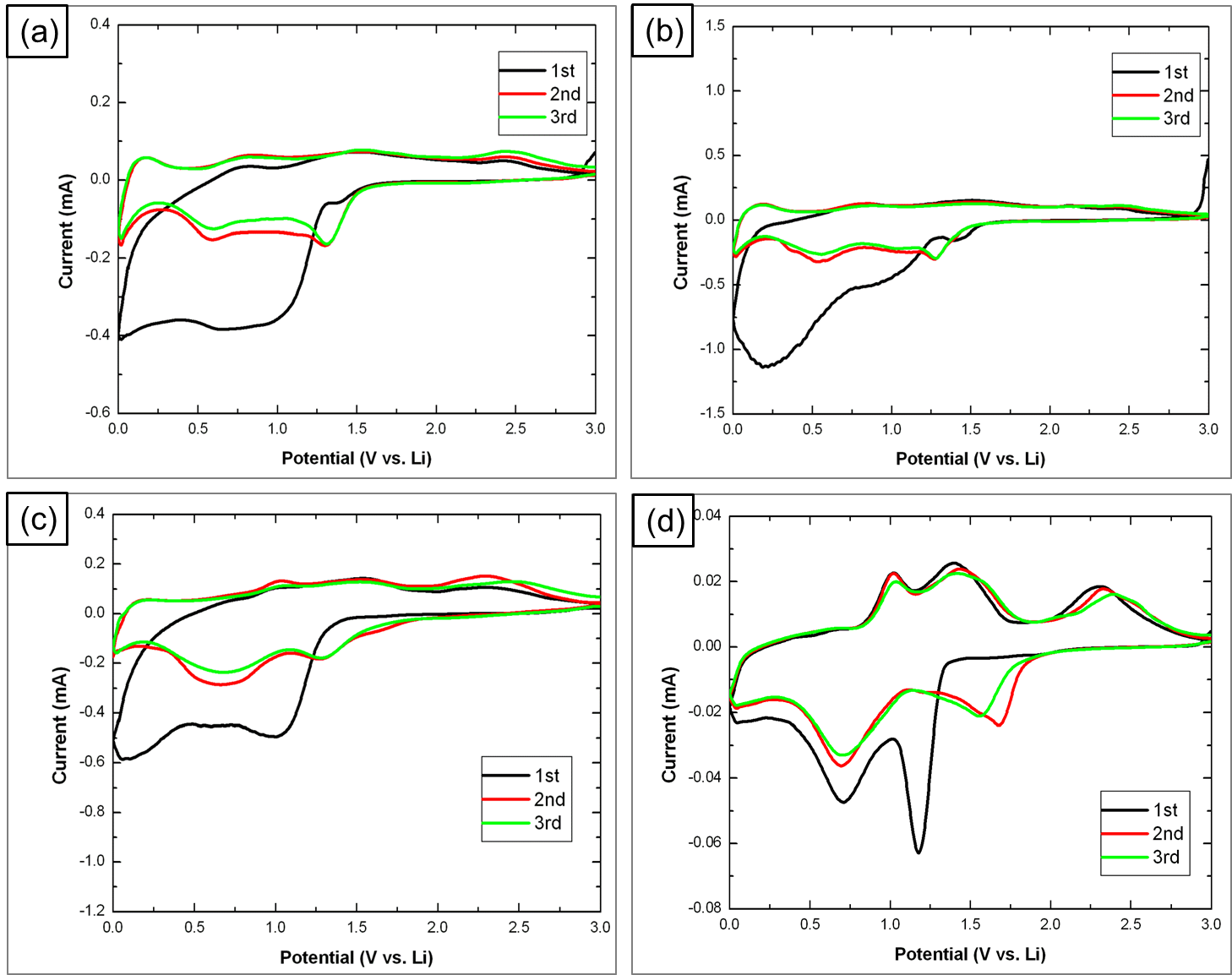


**Figure S6** CV curves of the CuO, CuO/NiO(Ni(NO_3_)_2_), CuO/NiO(NiSO_4_), and NiO(NiSO_4_) batteries.

**S7. The rate performances of CuO, CuO/NiO(Ni(NO_3_)_2_), CuO/NiO(NiSO_4_), and NiO(NiSO_4_)**

Fig. S7 shows the rate performances of the CuO, CuO/NiO(Ni(NO_3_)_2_), CuO/NiO(NiSO_4_), and NiO(NiSO_4_) batteries. The CuO/NiO(Ni(NO_3_)_2_) nanowires exhibit the highest capacity between the C-rate of 0.1C to 0.5C, but beyond 0.5C, the capacity is lower than that of sample CuO/NiO(NiSO_4_). This can be attributed to the better crystallinity of the CuO/NiO(NiSO_4_) nanowires. According to the diffraction patterns of Figs 6d and h, the complete reduction reaction of Cu can be achieved in the CuO/NiO(Ni(NO_3_)_2_) nanowire, but the center of the CuO/NiO(NiSO_4_) nanowire still remains as the CuO structure after lithiation. As the cycles of lithiation and delithiation proceed, the crystallinity of the CuO/NiO(Ni(NO_3_)_2_) nanowires will degrade and be converted into polycrystalline or amorphous. In contrast, the single crystal structure still remains in the core of the CuO/NiO(NiSO_4_) nanowire, so that its capacity retention is better during the high c-rate process.


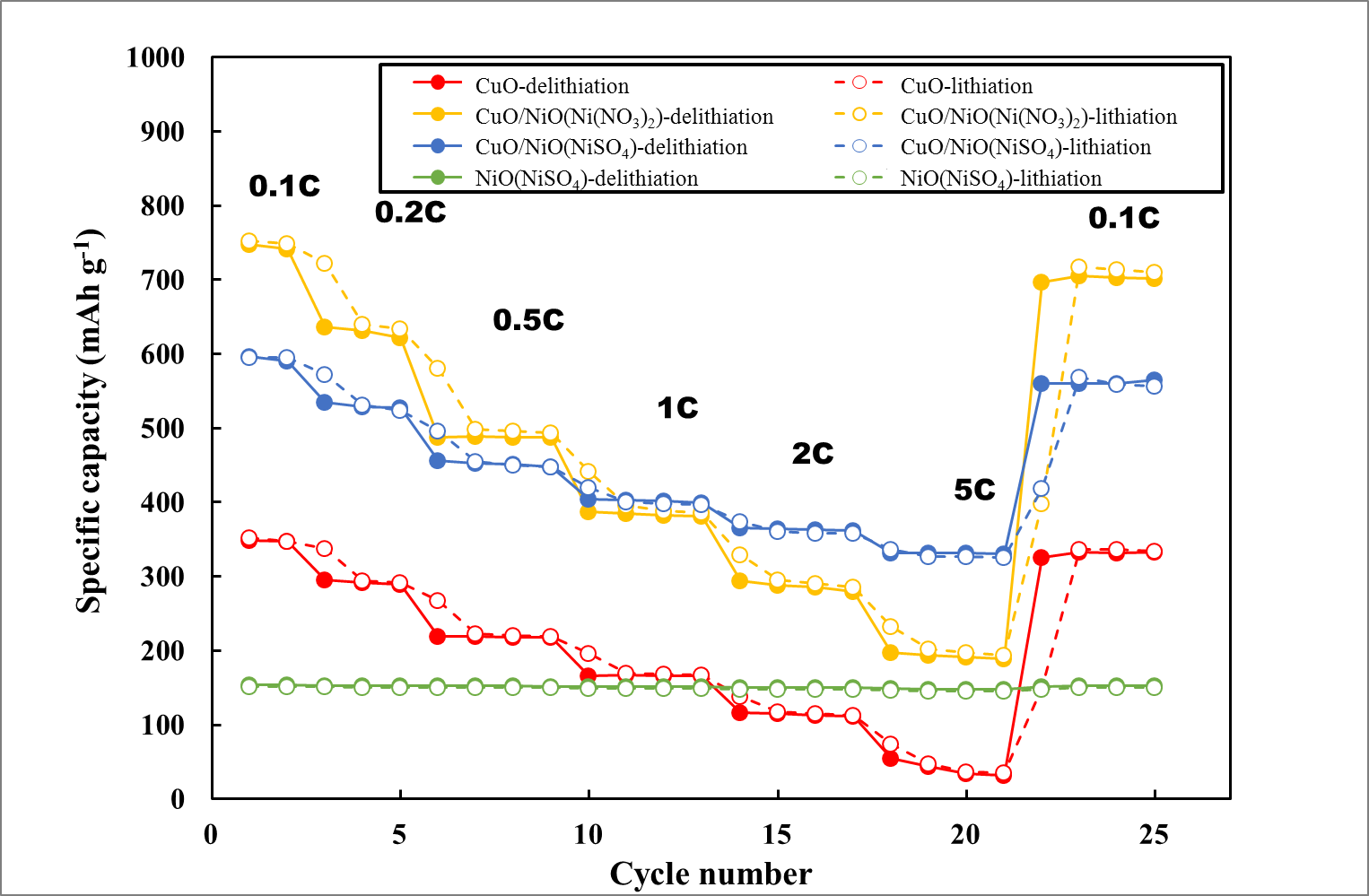


**Figure S7:** Rate performances of the CuO, CuO/NiO(Ni(NO_3_)_2_), CuO/NiO(NiSO_4_), and NiO(NiSO_4_) batteries.
